# The Self-Sorting Behavior of Circular Helicates and Molecular Knots and Links**

**DOI:** 10.1002/anie.201404270

**Published:** 2014-06-04

**Authors:** Jean-François Ayme, Jonathon E Beves, Christopher J Campbell, David A Leigh

**Affiliations:** J.-F. Ayme, Prof. D. A. Leigh School of Chemistry, University of ManchesterOxford Road, Manchester M13 9PL (UK); Dr. J. E. Beves, Dr. C. J. Campbell, Prof. D. A. Leigh School of Chemistry, University of Edinburgh, The King's BuildingsWest Mains Road, Edinburgh EH9 3JJ (UK)

**Keywords:** catenanes, chemical topology, helicates, molecular knots, supramolecular chemistry

## Abstract

We report on multicomponent self-sorting to form open circular helicates of different sizes from a primary monoamine, Fe^II^ ions, and dialdehyde ligand strands that differ in length and structure by only two oxygen atoms. The corresponding closed circular helicates that are formed from a diamine—a molecular Solomon link and a pentafoil knot—also self-sort, but up to two of the Solomon-link-forming ligand strands can be accommodated within the pentafoil knot structure and are either incorporated or omitted depending on the stage that the components are mixed.

The spontaneous segregation of molecular building blocks into discrete species within a mixture is known as self-sorting,^1^ a phenomenon that helps to maintain structural control over complex dynamic systems in nature.^2^ The use of orthogonal recognition elements is a convenient way to achieve sorting in artificial systems,^1^, ^3^ but other methods,^4^ including subtle differences in ligand design,^5^–^7^ can also be remarkably effective. A beautiful example is the classic experiment by Lehn and co-workers^5^ in which a mixture of ligand strands containing two to five 2,2′-bipyridine groups spontaneously self-sort into linear double helicates, each containing two ligands with equal numbers of binding sites, in the presence of Cu^I^ ions.

We recently described the synthesis of a molecular Solomon link^8^ (a doubly entwined [2]catenane^9^) and a molecular pentafoil knot,^10^ each formed through a combination of metal–ligand coordination, an anion template, and geometric restrictions. These closely related structures are derived from tetra-8 and pentameric^10^ circular helicate scaffolds, respectively, and are assembled from up to 20 common, or similar, components. Here we investigate the self-sorting behavior of both the closed molecular topologies and the open circular helicate scaffolds on which they are based (Figure 1). The study provides insights into the self-assembly processes of the individual species and reveals a subtle interplay between the driving forces and kinetic traps involved in their assembly.

**Figure 1:**
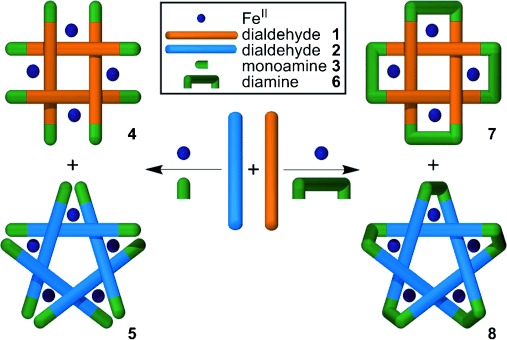
The assembly of circular helicates of different sizes and topologies from a primary amine (3) or diamine (6), Fe^II^ ions, and dialdehyde ligand strands (1 and 2).

Despite their structural similarities (a difference of just two oxygen atoms in length), dialdehydes **1** and **2** react individually with a suitable monoamine and FeCl_2_ to generate different-sized circular helicates: tetrameric^8^ with **1** and pentameric^10^ with **2**. To investigate the self-sorting potential of the ligands, a 1:1 mixture of aldehydes **1** and **2** was allowed to react with FeCl_2_ and *n*-hexylamine (**3**) in [D_6_]DMSO at 60 °C for 18 h, followed by anion exchange through the addition of an aqueous solution of potassium hexafluorophosphate (Scheme 1). ^1^H NMR spectroscopy (Figure 2 a, i) indicated the formation of both tetramer **4** and pentamer **5**, the spectrum of the reaction outcome being a superimposition of the spectra from the reaction of the individual aldehydes under similar experimental conditions (Figure 2 a, ii and iii). Electrospray mass spectrometry (ESIMS) confirmed perfect self-sorting, with no detectable formation of mixed-ligand species (Figure 2 b). Such fidelity is remarkable for such complex multicomponent systems made up from building blocks that vary only by a one-atom difference in the spacing of identical binding sites. The dynamics of this self-sorting system were further probed through experiments in which dialdehydes **1** and **2** were mixed at different points during the course of the reaction and monitored for up to four days at different concentrations (see the Supporting Information), which established that under these conditions the open circular helicates self-assemble and self-sort under thermodynamic control (see Section S2.1.4 in the Supporting Information for details).

**Scheme 1:**
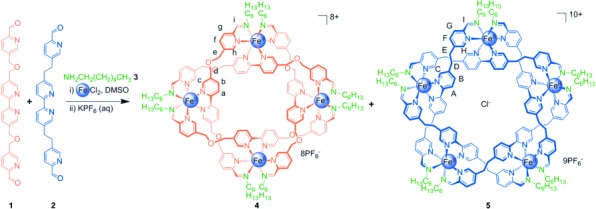
Perfect self-sorting of remarkably similar ligand strands in the formation of circular helicates of different sizes. A 1:1 ratio of aldehydes 1 and 2 was treated with two equivalents of FeCl_2_ and four equivalents of *n*-hexylamine (3) in [D_6_]DMSO at 60 °C for 18 h, followed by anion exchange with aqueous KPF_6_, thereby generating a mixture of circular helicates 4 and 5.

**Figure 2:**
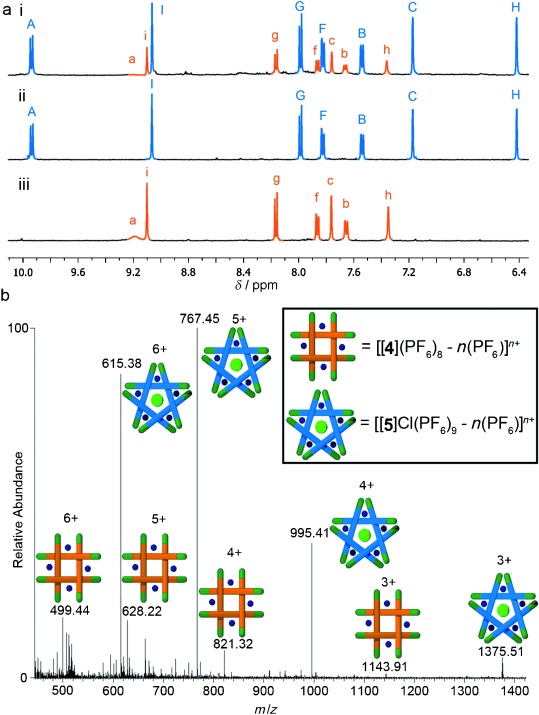
Spectroscopic analysis of the self-sorting reaction shown in Scheme 1. a) ^1^H NMR spectra (500 MHz, CD_3_CN, 298 K). i) The self-sorted mixture of open cyclic helicates 4 (orange) and 5 (blue), ii) pentameric cyclic helicate 5, and iii) tetrameric cyclic helicate 4. The broadness of the H^a^ signal is a function of chloride ion concentration.^8^ b) ESI mass spectrum of the self-sorted species shown in Scheme 1. Signals corresponding to helicates 4 and 5 with sequential loss of PF_6_ counterions are indicated.

The reaction of either aldehyde **1** or **2** with diamine **6** in the presence of Fe^II^ ions generates topological complex molecules:^11^ a Solomon link (four crossings arising from the tetrameric circular helicate scaffold)^8^ and pentafoil knot (five crossings arising from the pentameric circular helicate scaffold),^10^ respectively. However, the behavior of these closed circular helicate systems upon mixing was found to differ from that of the open analogues. The self-sorting experiment was conducted as previously, but with *n*-hexylamine substituted for 0.5 equiv of 2,2′-(ethylenedioxy)bis(ethylamine) (**6**) and the reaction times increased to four days (Scheme 2). After work up, the ^1^H NMR spectrum (Figure 3 a) showed two sets of signals corresponding to the formation of Solomon link **7** and pentafoil knot **8** accompanied by a series of low-intensity signals (shown in red in Figure 3 a). ESIMS analysis confirmed that the Solomon link is assembled almost exclusively from ligand **1**.^12^ However, in addition to pentafoil knot **8** (formed from five strands of ligand **2**), significant amounts of two other pentafoil knots, **9** and **10**, were present which arise from the incorporation of one or two strands of **1** into the pentafoil knot structure (see Figure S9 in the Supporting Information). The mixed-ligand-strand species pentafoil knot **9**, in which one strand of ligand **2** had been replaced with **1**, could be fully characterized by COSY and ROESY correlation experiments (see Figures S12–S14 in the Supporting Information) and is the main contributor to the low-intensity signals shown in red in Figure 3 a.^13^ Interestingly, the yield of Solomon link **7** in Scheme 2 remained unchanged relative to reactions in which only **2** was used (see Figure S15 in the Supporting Information), thus indicating that the mixed pentafoil knot species **9** and **10** arise principally at the expense of polymeric/oligomeric by-products rather than at the expense of the homoligand-strand pentafoil knot **8**. The product distribution was maintained over a range of concentrations (2–6 mm), with the relative yields of **7**, **8**, **9**, and **10** remaining constant throughout (see Figures S16–18 in the Supporting Information).

**Scheme 2:**
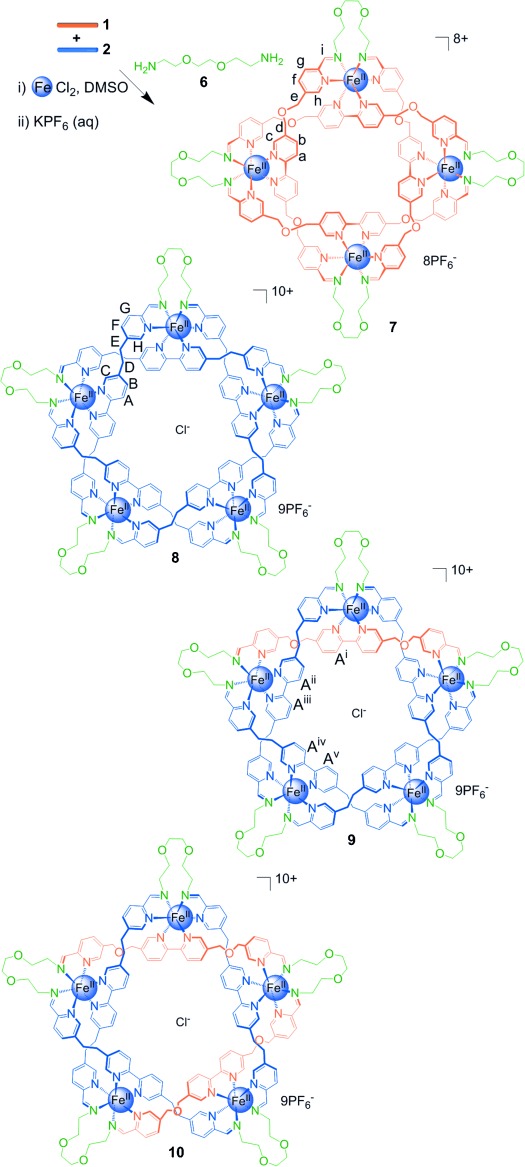
The assembly of knots and links using diamine 6. A 1:1 ratio of dialdehydes 1 and 2 was treated with two equivalents of FeCl_2_ and two equivalents of diamine 6 in [D_6_]DMSO at 60 °C for four days, followed by anion exchange with aqueous KPF_6_, to give Solomon link 7 and a mixture of pentafoil knots 8–10. Only one of the two isomers of 10 is shown (see Figure 4).

**Figure 3:**
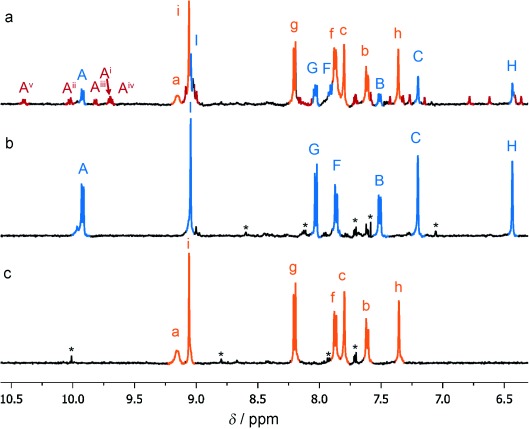
^1^H NMR spectra (500 MHz, CD_3_CN, 298 K). a) The mixture of Solomon link 7 (orange), pentafoil knot 8 (blue), mixed pentafoil knots 9 (red), and 10 (too small an amount to be visible by ^1^H NMR spectroscopy but observed using ESIMS; see Figure S10 in the Supporting Information) obtained by reaction of diamine 6 with dialdehydes 1 and 2. Products formed using only one dialdehyde: b) pentafoil knot 8 (from 2) and c) Solomon link 7 (from 1) prior to purification. The * marks small signals corresponding to aldehyde-containing ligand strands (products of imine hydrolysis) in (b) and (c).

To probe whether the distribution observed under the conditions employed in Scheme 2 is formed under thermodynamic control, two experiments were carried out that differed only in the time at which the dialdehydes were mixed (Figure 4). In the first experiment, dialdehydes **1** and **2** were mixed prior to the addition of amine **6** (Figure 4 a). In the second experiment, aldehydes **1** and **2** were allowed to react individually with diamine **6** (FeCl_2_, [D_6_]DMSO, 60 °C) for 24 h prior to combining both reactions (Figure 4 b). The resulting mixtures were heated at 60 °C and the change in the product distribution monitored over seven days. If compounds **7**–**10** are under thermodynamic control, then both experimental procedures should equilibrate to the same distribution (as is observed with the monoamine-derived circular helicates (Scheme 1) and see Section S2.1.4 in the Supporting Information). However, the outcomes of the two experiments involving the diamine are very different (Figure 5). When the dialdehydes are combined from the start, the mixed-ligand-strand pentafoil knots **9** and **10** are formed (in addition to **7** and **8**) as expected (Figure 5 a). In contrast, when the aldehydes are allowed to react individually with diamine **6** and FeCl_2_ for 24 h and then the reaction mixtures (which include not only some of the closed cyclic helicates, but also oligomers and polymeric by-products) are heated further, there is no evidence of mixed-ligand species even after seven days (Figure 5 b).

**Figure 4:**
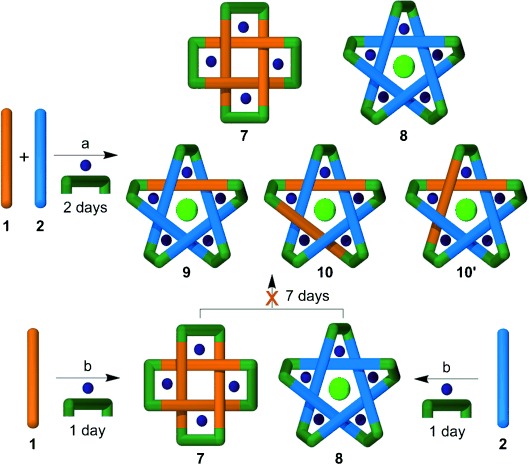
Assembly of molecular Solomon link 7 and pentafoil knots 8–10 using different experimental procedures. The product distribution of the closed topologies is dependent on when the reaction mixtures are combined.

**Figure 5:**
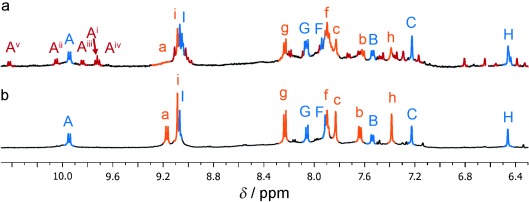
^1^H NMR spectra (500 MHz, CD_3_CN, 298 K). a) Reaction mixture from Figure 4 a (after PF_6_^−^ ion exchange), where aldehydes 1 and 2 are mixed prior to the addition of diamine 6, showing significant amounts of mixed-ligand pentafoil knot 9 (red). b) Reaction mixture from Figure 4 b (after PF_6_^−^ ion exchange), where preformed 7 (orange) and 8 (blue) were mixed, shows no indication of the presence of mixed-ligand pentafoil knots.

Clearly, under these conditions (60 °C, 7 days) this system is not under thermodynamic control. The mixed-ligand-strand pentafoil knots **9** and **10** are kinetic products, similar in accessibility to **8**. The rationale for the differing behavior of the open helicates and the closed molecular topologies is the relative ease of dissociation of the different types of ligands. In the open systems (**4** and **5**), the exchange of ligand units involves only metal–ligand dissociation of a single tris(bidentate) strand, which is sufficiently rapid for equilibrium to be reached under the reaction conditions. However, unless ligand exchange occurs by hydrolysis, then for a tris(bidentate) strand to be replaced in the closed systems the two neighboring strands also have to dissociate from iron centers for imine exchange of the diamine linker to occur. The energy cost of this additional process is evidently too high to allow efficient rearrangement of **9** and **10**, thereby preventing the closed systems from undergoing full “error-checking” under thermodynamic control.^16^

In conclusion, the reaction of **1** and **2** with *n*-hexylamine (**3**) leads to a perfectly self-sorted and dynamic mixture of open circular helicates of different sizes, **4** and **5**. Although this involves formation of imine bonds, it is effectively a cyclic version of the self-sorting experiment with linear helicates pioneered by Lehn and co-workers,^5^ but instead of using ligand strands that sort according to the number of bidentate binding sites and overall length, **1** and **2** have the same number of binding sites and differ only by a one atom spacing of those binding sites within the strand.^14^, ^15^ Nonetheless, each ligand is able to effectively distinguish self from non-self in forming different-sized circular assemblies and the components are able to exchange in-and-out of the circular helicates in a facile manner. Dialdehydes **1** and **2** also largely self-sort according to the size of the circular helicate in their reaction with diamine **6**, thereby generating Solomon link **7** and pentafoil knot **8**, respectively. In this case, however, the self-sorting is imperfect and mixed-ligand-strand pentafoil knots **9** and **10** are also formed. The fully closed circular helicates do not readily exchange their ligand strands even over extended reaction times.

These systems illustrate not only the exquisite fidelity that is possible in the self-sorting of very similar building blocks within complex multicomponent assemblies, but also how the same modest differences in structure can tip the balance between thermodynamic control and kinetic trapping. Learning how to recognize, understand, and, ultimately, manipulate such processes will be an important step towards mimicking nature’s mastery of molecular assembly with synthetic systems.
