# Supplementary material for: The Self-Sorting Behavior of Circular Helicates and Molecular Knots and Links
Source: Angew Chem Int Ed Engl. 2014 Jun 4;53(30):7823–7. doi: 10.1002/anie.201404270 (PMC4321334; doi:10.1002/anie.201404270)
Supplement: Supplementary file 1 [file anie0053-7823-sd1.pdf]

Angewandte Chemie
Eine Zeitschrift der Gesellschaft Deutscher Chemiker

Supporting Information

© Wiley-VCH 2014

69451 Weinheim, Germany

The Self-Sorting Behavior of Circular Helicates and Molecular Knots and Links**

*Jean-François Ayme, Jonathon E. Beves, Christopher J. Campbell, and David A. Leigh**

anie_201404270_sm_miscellaneous_information.pdf

Supporting Information

1. General Experimental S1
2. Self-Sorting Reactions S3
   2.1 Open Systems S3
   2.1.1 Experimental Procedure for the Self-Sorting of Cyclic Helicates **4** and **5** S3
   2.1.2 Experimental Procedure to Determine the Effects of Mixing on the Relative Yields of Cyclic Helicates **4** and **5** S4
   2.1.3 Experimental Procedure to Determine the Effects of Concentration on the Self-Sorting of Helicates **4** and **5** S5
   2.1.4 Thermodynamic Investigations of the Self-Sorting of Helicates **4** and **5** S7
   2.2 Closed Systems S11
   2.2.1 Experimental Procedure for the Self-Sorting of Solomon Link **7** and Pentafoil Knot **8** S11
   2.2.2 Experimental Procedure to Determine the Effects of Mixing on the Relative Yields of Solomon Link **7** and Pentafoil Knot **8** S15
   2.2.3 Experimental Procedure to Determine the Effects of Concentration on the Self-Sorting of Closed Topologies **7**, **8**, **9** and **10** S17
   2.2.4 Experimental Procedure For the Thermodynamic Investigations of the Self-Sorting of Closed Topologies **7**, **8**, **9** and **10** S19
3. References S20

1. General Experimental

Unless stated otherwise, all reagents and solvents were purchased from Aldrich Chemicals and used without further purification. NMR spectra were recorded on a Bruker DMX 500 instrument. Chemical shifts are reported in parts per million (ppm) from low to high frequency and referenced to the residual solvent resonance. Coupling constants (*J*) are reported in hertz (Hz). Standard abbreviations indicating multiplicity were used as follows: s = singlet, d = doublet, t = triplet, dd = double doublet, q = quartet, m = multiplet, b = broad, ddd = doublet of double doublets. ^{1}H and ^{13}C NMR assignments were made using 2D-NMR methods (COSY, ROESY, HSQC, HMBC) and are unambiguous unless stated otherwise. Low resolution ESI mass spectrometry was performed with a Finnigan LCQ-MS, Micromass Platform II or Waters Quattro Ultima LC-*MS*/*MS* mass spectrometers. High resolution ESI mass spectrometry was carried out by the mass spectrometry services at the Universities of Edinburgh and Manchester and the EPSRC National Mass Spectrometry Service Centre, Swansea, UK. Aldehydes **1**[S1] and **2**[S2] were prepared according to literature procedures.

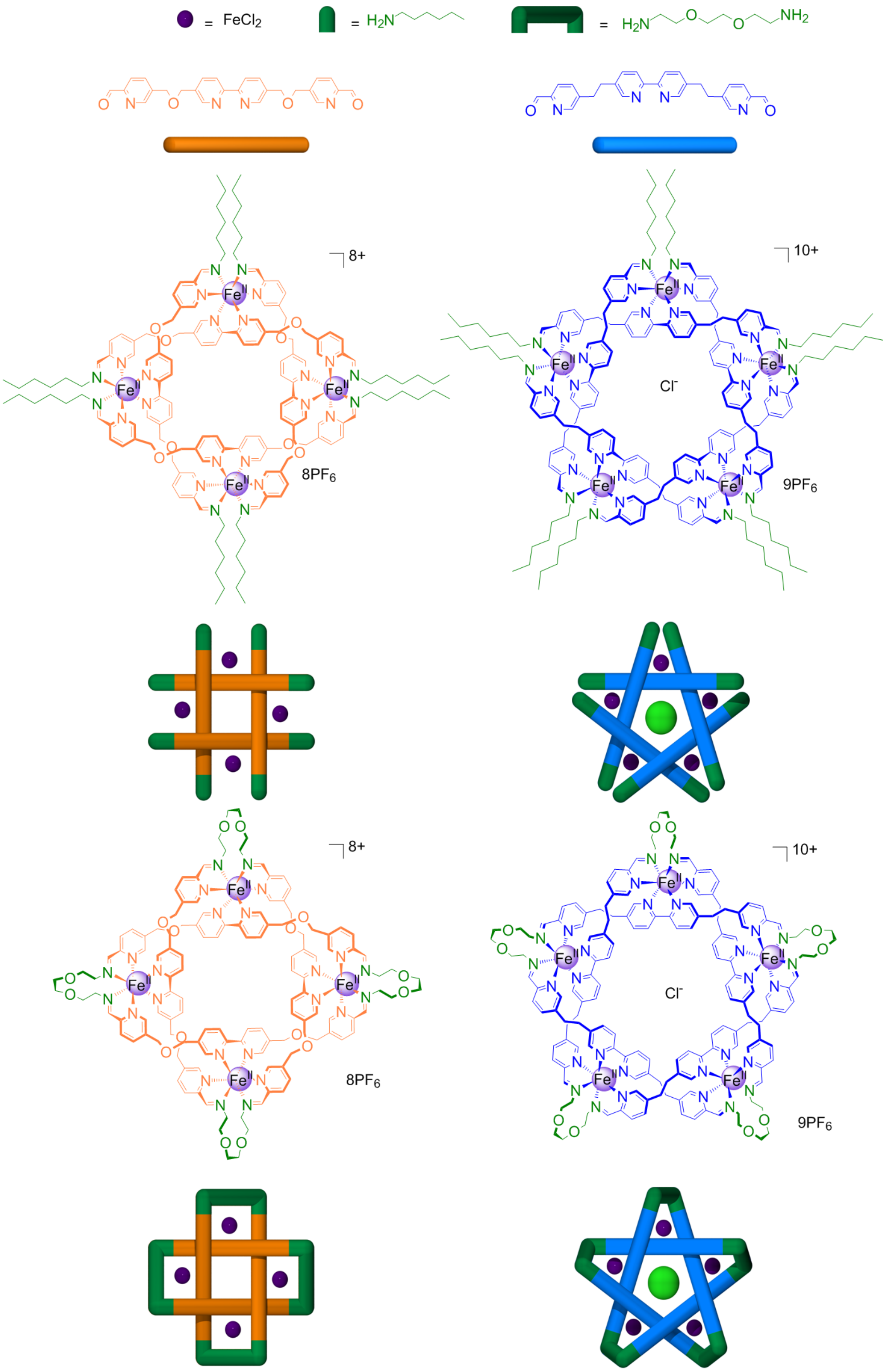

Figure S1. Structural representations of the cartoons used throughout the manuscript and supporting information.

2. Self-Sorting Reactions

Experiments are described in two sections, dealing with the "open" cyclic helicates **4** and **5** first, followed by the "closed" topologically complex systems **7** and **8**.

2.1 Open Systems

2.1.1 Experimental Procedure for the Self-Sorting of Cyclic Helicates 4 and 5

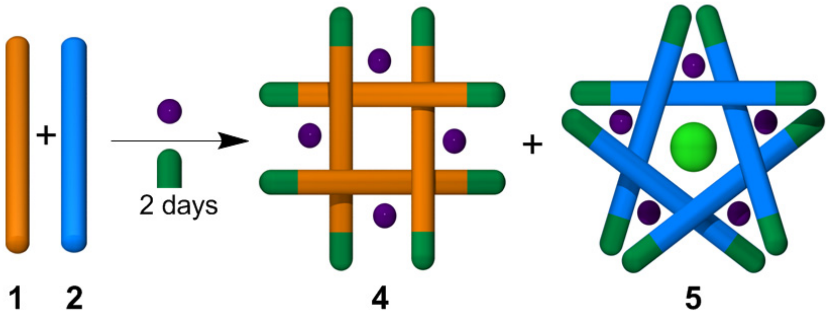

Scheme S1. Mixing experiments conducted with aldehyde **1** (orange) and aldehyde **2** (blue) with iron(II) (purple) and hexylamine (green) to form tetramer **4** and pentamer **5**.

To two separate DMSO-d_6 solutions of **1** (1.1 mg, 2.4 µmol, in 0.5 mL) and **2** (1.0 mg, 2.4 µmol in 0.5 mL) was added $FeCl_2$ (25 µL of a 0.10 M stock solution, 2.6 µmol). The two purple solutions were mixed thoroughly to ensure complete dissolution of both dialdehydes. The solutions were combined together in a clean NMR tube before addition of *n*-hexylamine (50 µL of a 0.21 M stock solution, 5.2 µmol). The resulting mixture was heated for 2 d at 60 ºC before being allowed to cool. The reaction mixture was precipitated using KPF_6 (saturated aqueous solution). The resulting purple powder was collected on Celite and washed with water, a small amount of EtOH (**4** is soluble) and finally Et_2O. The product mixture was dissolved in CH_3CN and the solvent removed under reduced pressure to give a mixture of **4** and **5** as a purple powder. The mixture was fully soluble in CH_3CN and could be analyzed by ^{1}H NMR and LR-ESI (Figure 1, main text). All results were consistent with previous reports for **4**[S1] and **5**[S2].

2.1.2 Experimental Procedure to Determine the Effects of Mixing on the Relative Yields of Cyclic Helicates 4 and 5

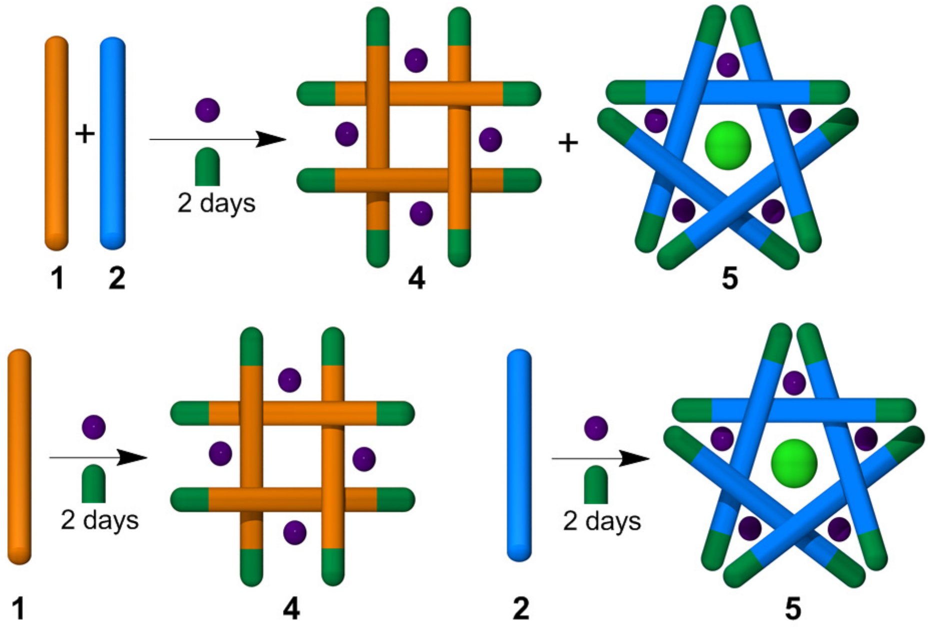

Scheme S2. Self-sorting experiments conducted to determine the effect of mixing on the relative yields of **4** and **5**. Reactions were carried out using stock solutions of dialdehydes **1** and **2** under identical conditions.

To two separate DMSO-d_6 solutions of **1** (2.1 mg, 4.7 μmol, in 1.0 mL) and **2** (2.0 mg, 4.7 μmol in 1.0 mL) was added $FeCl_2$ (50 μL of a 0.10 M stock solution, 5.2 μmol). The two purple solutions were mixed thoroughly to ensure complete dissolution of both dialdehydes. A 0.5 mL aliquot of **1**+$FeCl_2$ was mixed with a 0.5 mL aliquot of **2**+$FeCl_2$ in a clean NMR tube before addition of *n*-hexylamine (50 μL of a 0.208 M stock solution, 5.2 μmol). The remaining 0.5 mL aliquots of **1**+$FeCl_2$ and **2**+$FeCl_2$ were separately reacted with *n*-hexylamine (25 μL of a 0.21 M stock solution, 5.2 μmol, for each reaction). The three solutions were heated for 2 d at 60 ºC before being allowed to cool. The following purification procedure was applied to each sample individually: The reaction mixture was precipitated using KPF_6 (saturated aqueous solution). The resulting purple powder was collected on Celite and washed with water, a small amount of EtOH (**4** is soluble) and finally Et_2O. The product mixture was dissolved in CH_3CN and the solvent removed under reduced pressure. The three samples were dissolved in 0.5 mL of CD_3CN and compared by ^1H NMR (Figure S2).

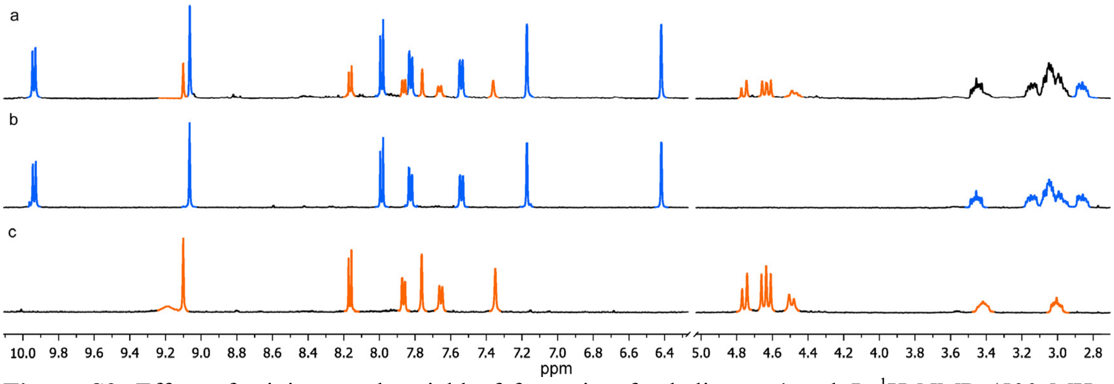

Figure S2. Effect of mixing on the yield of formation for helicates **4** and **5**. ^{1}H NMR (500 MHz, CD_3CN), (a) self-sorted mixture of **4** (orange) and **5** (blue), (b) **5** only, (c) **3** only. Reactions were run at the same concentration with dialdehydes taken from a stock solution. As can be seen the yield of formation of pentamer **5** is unaffected by the presence of **4** whereas the yield of **4** is reduced by 50 % upon mixing.

2.1.3 Experimental Procedure to Determine the Effects of Concentration on the Self-Sorting of Helicates 4 and 5

To two separate DMSO-d_6 solutions of **1** (6.4 mg, 14.2 µmol, in 2.4 mL) and **2** (6.0 mg, 14.2 µmol in 2.4 mL) was added $FeCl_2$ (150 µL of a 0.10 M stock solution, 15.6 µmol). The two purple solutions were mixed thoroughly to ensure complete dissolution of both dialdehydes. Three mixed samples were analyzed differing in the concentration of the reaction mixture.

Sample 1, concentration 5.9 mM:

A 0.4 mL aliquot of **1**+$FeCl_2$ was mixed with a 0.4 mL aliquot of **2**+$FeCl_2$ in a clean NMR tube before addition of *n*-hexylamine (50 µL of a 0.208 M stock solution, 5.2 µmol). 0.4 mL aliquots of **1**+$FeCl_2$ and **2**+$FeCl_2$ were separately reacted with *n*-hexylamine (25 µL of a 0.21 M stock solution, 5.2 µmol, for each reaction).

Sample 2, concentration 4.7 mM:

A 0.4 mL aliquot of **1**+$FeCl_2$ was mixed with a 0.4 mL aliquot of **2**+$FeCl_2$ in a clean NMR tube before addition of DMSO-d_6,(0.6 mL) then *n*-hexylamine (50 µL of a 0.208 M stock solution, 5.2 µmol). 0.4 mL aliquots of **1**+$FeCl_2$ and **2**+$FeCl_2$ were separately diluted with DMSO-d_6 (0.1 mL) then reacted with *n*-hexylamine (25 µL of a 0.21 M stock solution, 5.2 µmol, for each reaction).

Sample 3, concentration 2.4 mM:

A 0.4 mL aliquot of **1**+$FeCl_2$ was mixed with a 0.4 mL aliquot of **2**+$FeCl_2$ in a clean NMR tube before addition of DMSO-d_6,(1.2 mL) then *n*-hexylamine (50 µL of a 0.208 M stock solution, 5.2 µmol). 0.4 mL aliquots of **1**+$FeCl_2$ and **2**+$FeCl_2$ were

separately diluted with DMSO-d_6 (0.6 mL) then reacted with *n*-hexylamine (25 μL of a 0.21 M stock solution, 5.2 μmol, for each reaction).

All nine reactions were heated for 1 d at 60 ºC before being allowed to cool. The following purification procedure was applied to each sample individually: The reaction mixture was precipitated using KPF_6 (saturated aqueous solution). The resulting purple powder was collected on Celite and washed with water, a small amount of EtOH (**4** is soluble) and finally Et_2O. The product mixture was dissolved in CH_3CN and the solvent removed under reduced pressure. The nine samples were dissolved in 0.5 mL of CD_3CN and compared by ^{1}H NMR (Sample 1, Figure S3. Sample 2, Figure S4. Sample 3, Figure S5).

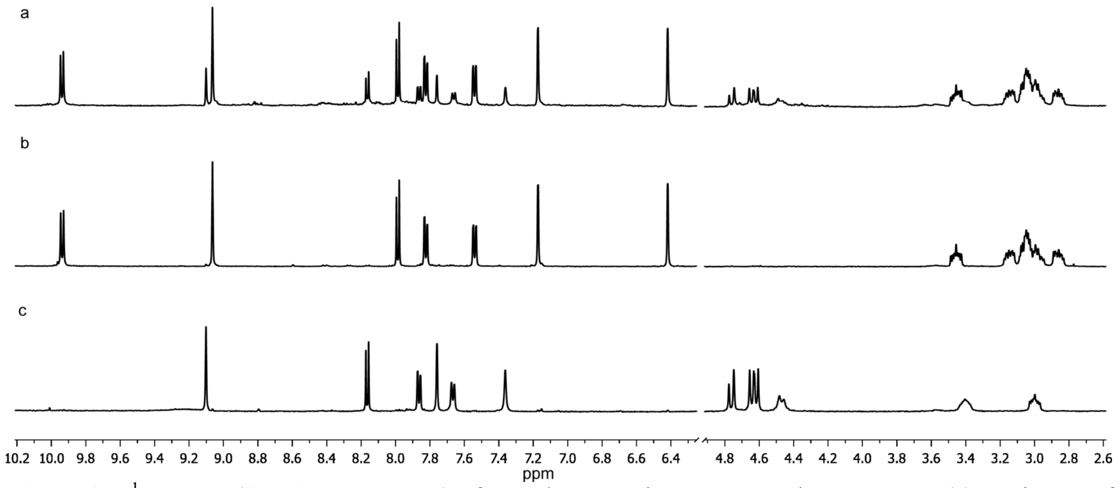

Figure S3. ^{1}H NMR (CD_3CN, 500 MHz) of sample 1 reaction concentration; 5.9 mM. (a) a mixture of **4** and **5**, (b) **5** and (c) **4**.

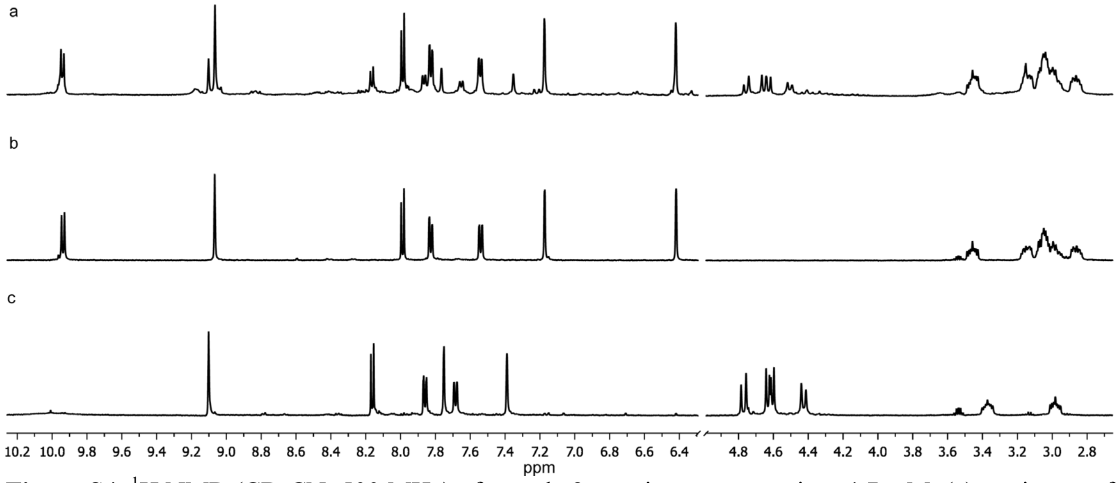

Figure S4. ^{1}H NMR (CD_3CN, 500 MHz) of sample 2 reaction concentration; 4.7 mM. (a) a mixture of **4** and **5**, (b) **5** and (c) **4**.

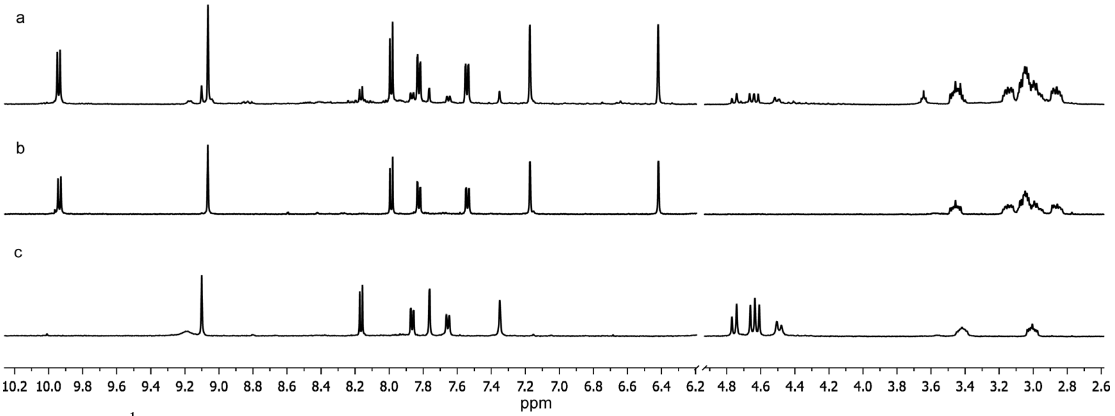

Figure S5. ^1H NMR (CD_3CN, 500 MHz) of sample 3 reaction concentration; 2.4 mM. (a) a mixture of **4** and **5**, (b) **5** and (c) **4**.

2.1.4 Thermodynamic Investigations of the Self-Sorting of Helicates 4 and 5

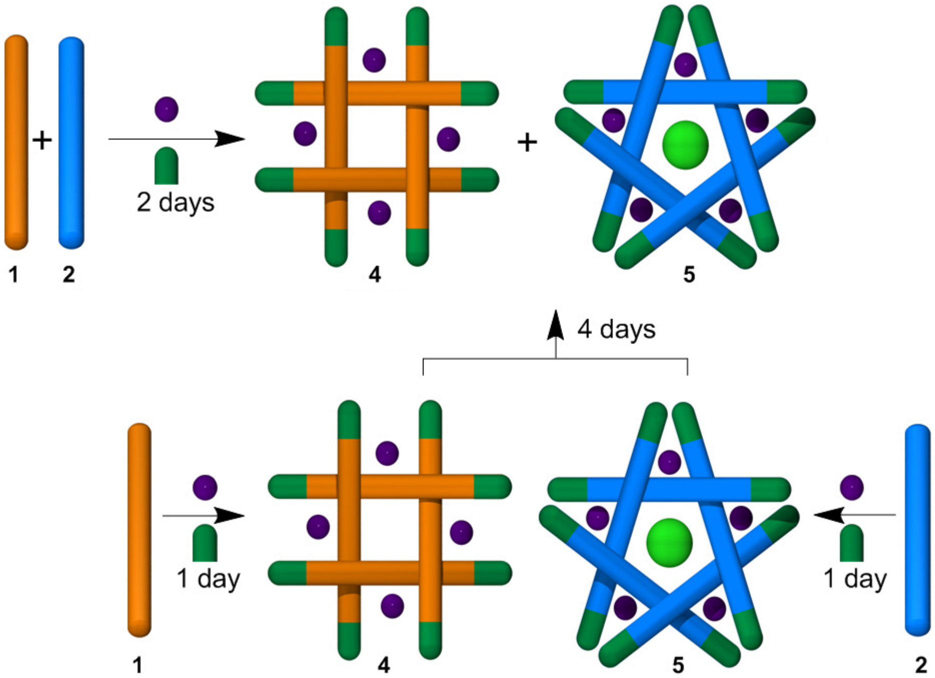

Scheme S3. Thermodynamic investigations. The products of the mixing reaction are the same regardless of the time at which dialdehydes **1** and **2** are mixed.

The thermodynamics of self-sorting was probed by monitoring reactions C and D mixed at different times during the self-assembly process (Scheme S3). Two samples were prepared (see below for details); In sample C aldehydes **1** and **2** (with $FeCl_2$) were mixed prior to the addition of *n*-hexylamine **3** and sample D where each dialdehyde (with $FeCl_2$) was reacted separately with *n*-hexylamine, then after 24h these reaction mixtures were combined. Both samples were heated at 60 ºC and monitored over the course of 4 days (Figure S6). As expected after the first day of heating sample C shows the required distribution of **4** and **5**, which remains constant

over the course of 4 d (see Figure S6d). Immediately after mixing sample D shows an approximately 1:1 distribution of helicates **4** and **5** (Figure S7a), which slowly rearranges to the ~1:3 ratio seen above (see Figure S7d). Upon work up both samples C and D appear to be identical (see Figures S7a and b) therefore both reactions reach the same end point irrespective of the times mixed. Indicating that the formation of **4** is dynamic (as its abundance was observed to decrease) and that it is possible to disassemble preformed tetrameric helicate **4** by addition of the reaction mixture for the formation of **5**.

2.1.4.1 Experimental Procedure for the Thermodynamic Investigations of the Self-Sorting of Helicates 4 and 5

To two separate DMSO-d_6 solutions of **1** (2.1 mg, 4.7 µmol, in 1.0 mL) and **2** (2.0 mg, 4.7 µmol in 1.0 mL) was added $FeCl_2$ (50 µL of a 0.10 M stock solution, 5.2 µmol). The two purple solutions were mixed thoroughly to ensure complete dissolution of both dialdehydes. A 0.5 mL aliquot of **1**+$FeCl_2$ was mixed with a 0.5 mL aliquot of **2**+$FeCl_2$ in a clean NMR tube before addition of *n*-hexylamine (50 µL of a 0.21 M stock solution, 5.2 µmol) (sample C). The remaining 0.5 mL aliquots of **1**+$FeCl_2$ and **2**+$FeCl_2$ were separately reacted with *n*-hexylamine **3** (25 µL of a 0.21 M stock solution, 5.2 µmol, for each reaction). The three solutions were heated for 1 d at 60 ºC. The individual solutions of **1**+$FeCl_2$+**3** and **2**+$FeCl_2$+**3** were combined in a clean NMR tube and thoroughly mixed before being heated at 60°C for a further 4 days (sample D). Both samples C and D were monitored over this time (Figures S6 and S7). The two samples were allowed to cool and the following purification procedure was applied to each sample individually: The reaction mixture was precipitated using KPF_6 (saturated aqueous solution). The resulting purple powder was collected on Celite and washed with water, a small amount of EtOH (**4** is soluble) and finally Et_2O. The product mixture was dissolved in CH_3CN and the solvent removed under reduced pressure. The two samples were dissolved in 0.5 mL of CD_3CN compared by ^1H NMR (Figure S8).

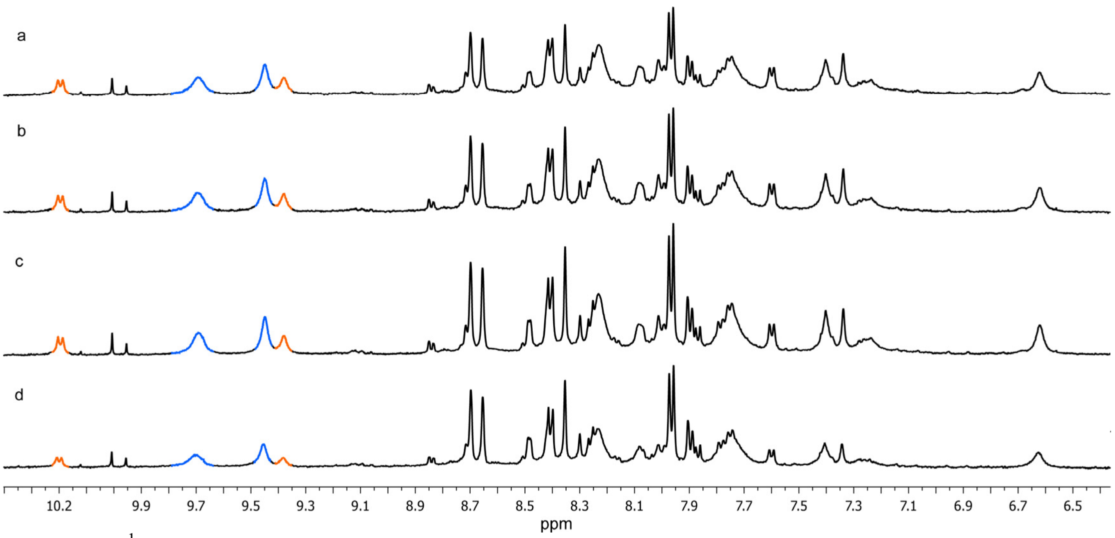

Figure S6. ^{1}H NMR (DMSO-d_6, 500 MHz) analysis over time of the crude reaction mixture of solution C for the formation of **4** (orange) and **5** (blue) where dialdehydes **1** and **2** were mixed at the start of the reaction. Spectra correspond to the reaction mixture after (a) 1 day (b) 2 days, (c) 3 days, (d) 4 days. The ratio of **4:5** remains constant over the course of the experiment.

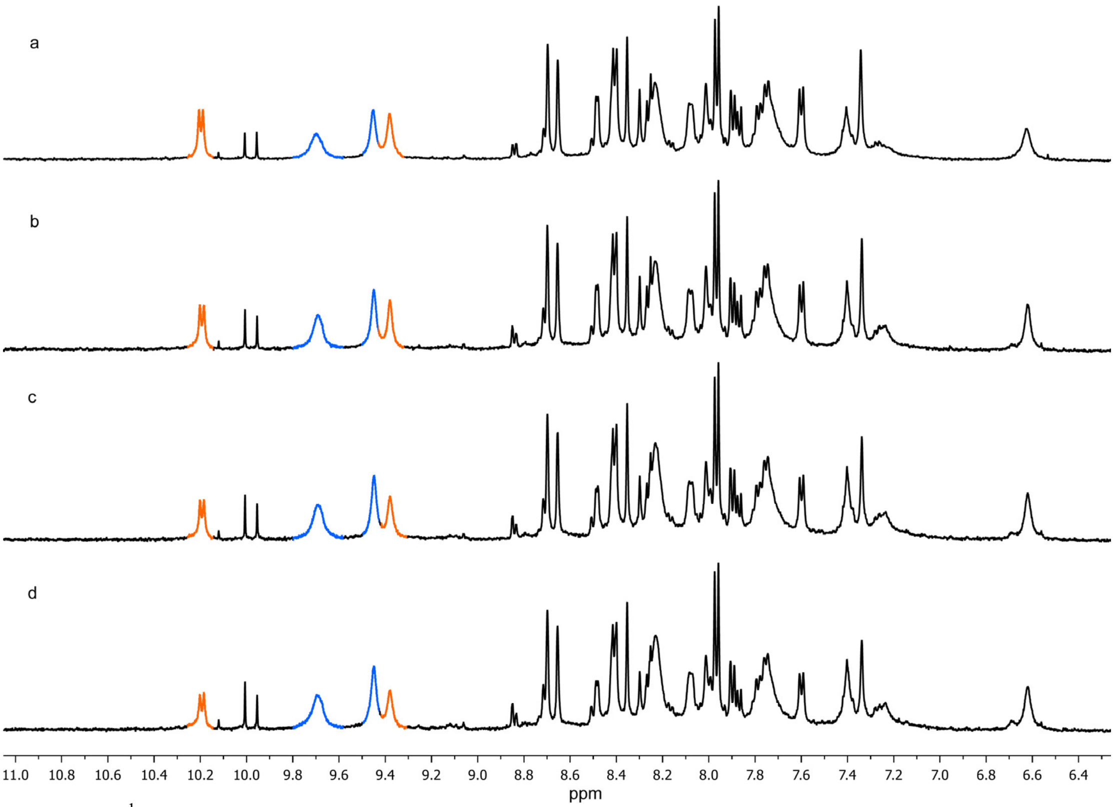

Figure S7. ^{1}H NMR (DMSO-d_6, 500 MHz) analysis over time of the crude reaction mixture of solution D the formation of **4** (orange) and **5** (blue) where dialdehydes **1** and **2** were mixed after 1 d. Spectra correspond to the reaction mixture (a) immediately after mixing (b) after 1 day, (c) 2 days, (d) 3 days. The ratio of **4**:**5** starts at 1:1 as expected for mixing equimolar solutions of **4** and **5**. Over time the yield of **5** is depleted until after 4 days of heating the ratio matches that in Figure S6.

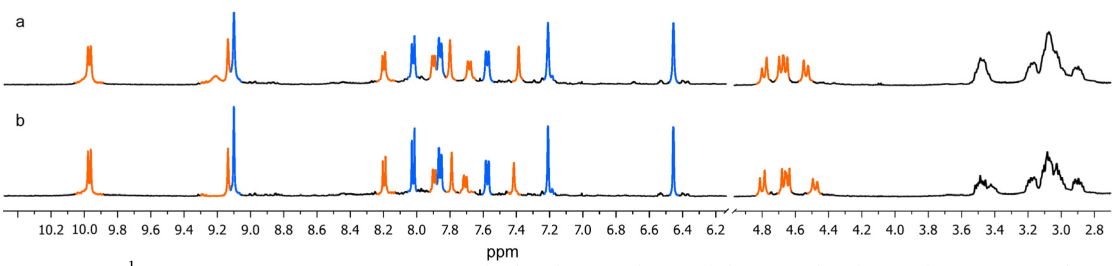

Figure S8. ^{1}HNMR (CD_3CN, 500 MHz) analysis after work up of the samples from Figures S6 and S7. **4** (orange) and **5** (blue) (a) solution C, (b) solution D.

2.2 Closed Systems

2.2.1 Experimental Procedure for the Self-Sorting of Solomon Link 7 and Pentafoil Knot 8

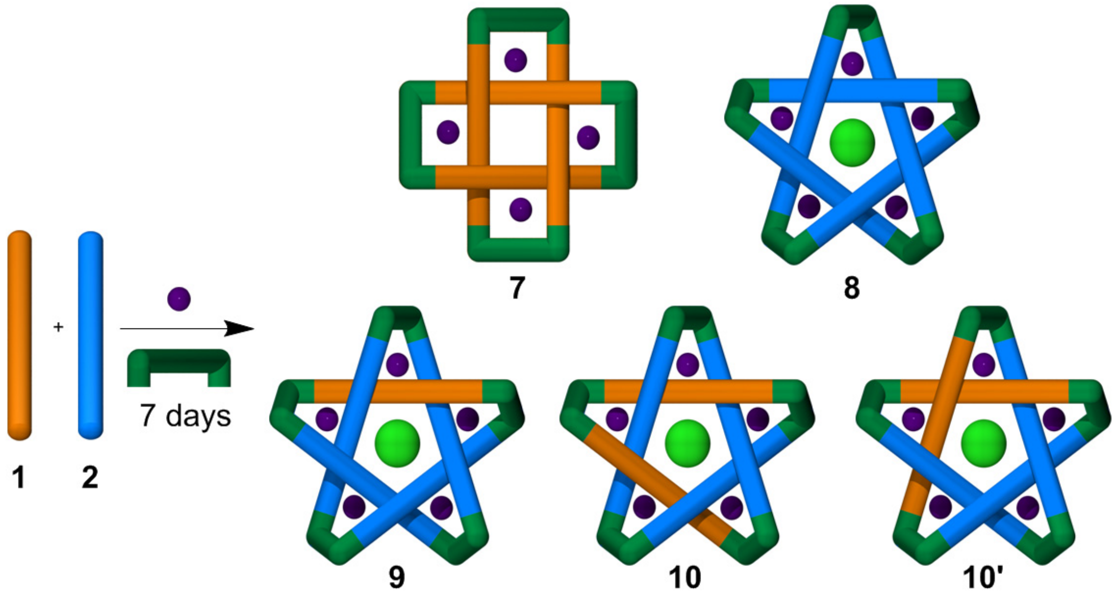

Scheme S4. Mixing experiments conducted with aldehyde **1** (orange) and aldehyde **2** (blue) with iron(II) (purple) and 2,2'-(ethylenedioxy)bis(ethylamine) (green) to form Solomon link **7** and pentafoil knots **8**, **9** and **10**.

To two separate solutions of **1** (1.1 mg, 2.4 μmol, in 0.5 mL) and **2** (1.0 mg, 2.4 μmol in 0.5 mL) was added a solution of anhydrous $FeCl_2$ (25 μL of a 0.104 M stock solution, 2.6 μmol). The two purple solutions were mixed thoroughly to ensure complete dissolution of both dialdehydes. The solutions were combined together in a clean NMR tube before addition of a solution of 2,2'-(ethylenedioxy)bis(ethylamine) (50 μL of a 0.10 M stock solution, 2.6 μmol). The resulting mixture was heated for 7d at 60 ºC before being allowed to cool to RT. The reaction mixture was precipitated using an excess saturated aqueous KPF_6. The resulting purple powder was collected on Celite and washed with water, a small amount of EtOH and finally Et_2O. The product mixture was dissolved in CH_3CN and the solvent removed under reduced pressure to give a mixture of **6**, **7**, **8** and **9** as a purple powder which was analyzed by ^{1}H NMR (Figure 2, main text), LR-ESI (Figure S9), HR-ESI (Figures S10 and 11), COSY (Figure S12) and ROESY (Figures S13 and 14) spectroscopy.

2.2.1.1 Characterization of Closed Topologies 7, 8 9 and 10

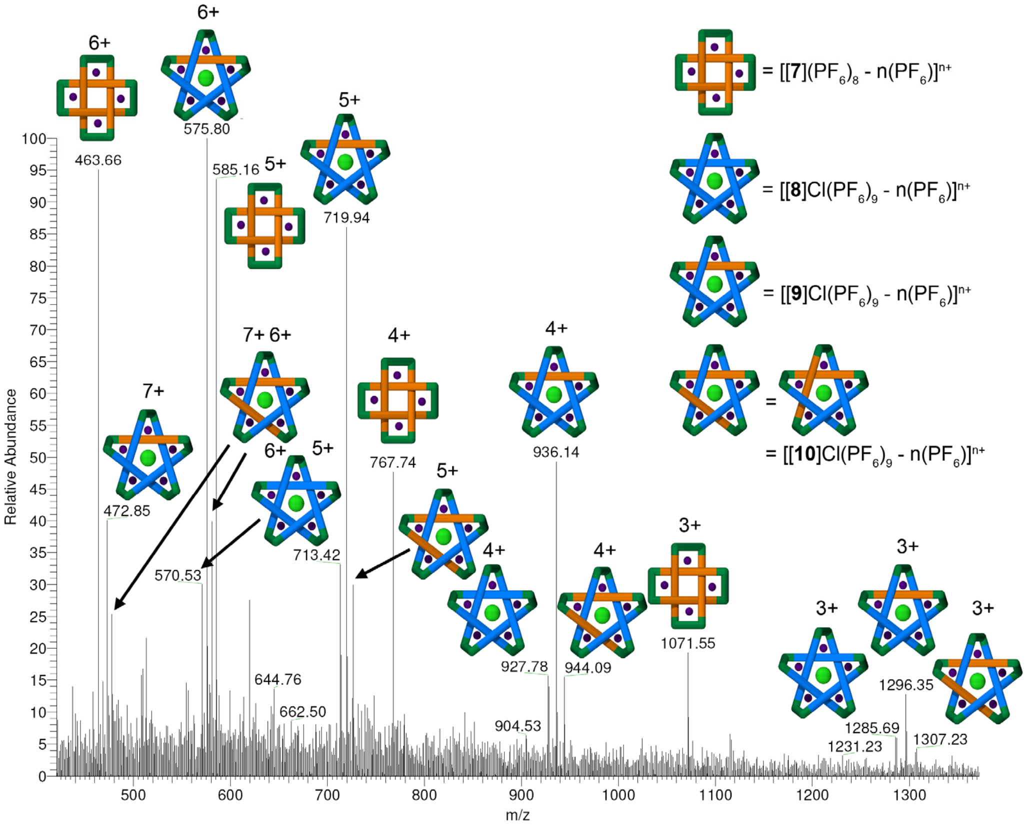

Figure S9. LRESI-MS analysis of the mixture of **7**, **8**, **9** and **10**.

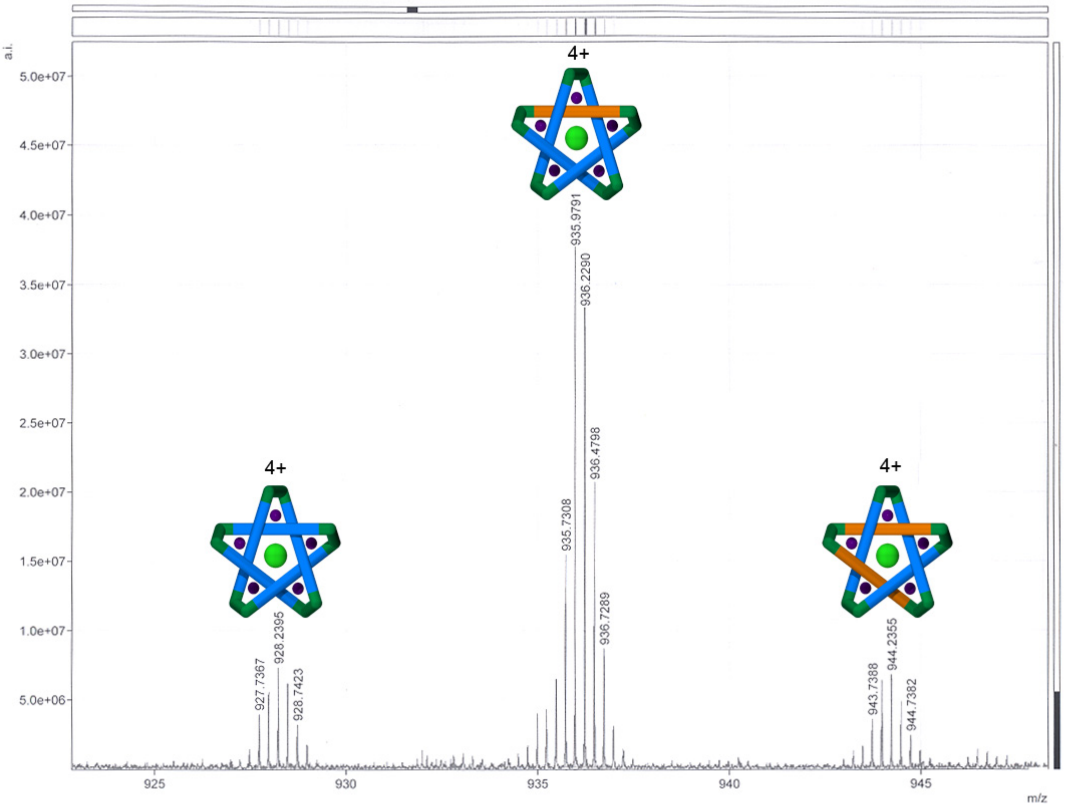

Figure S10. HRESI-MS highlighting pentafoil knots **7**, **8** and **9**.

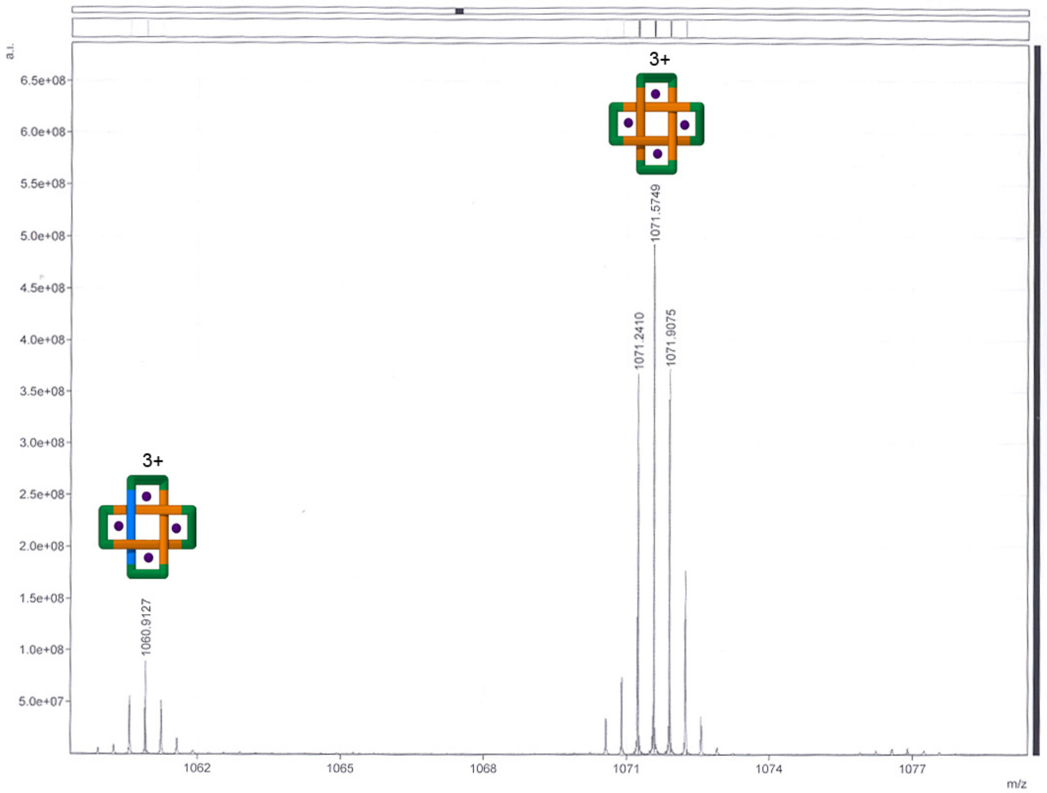

Figure S11. HRESI-MS highlighting Solomon link **4** and a mixed Solomon link incorporating one alternative component **2**.

2.2.1.2 2D NMR Characterisation of Mixed-Ligand Pentafoil Knot 9

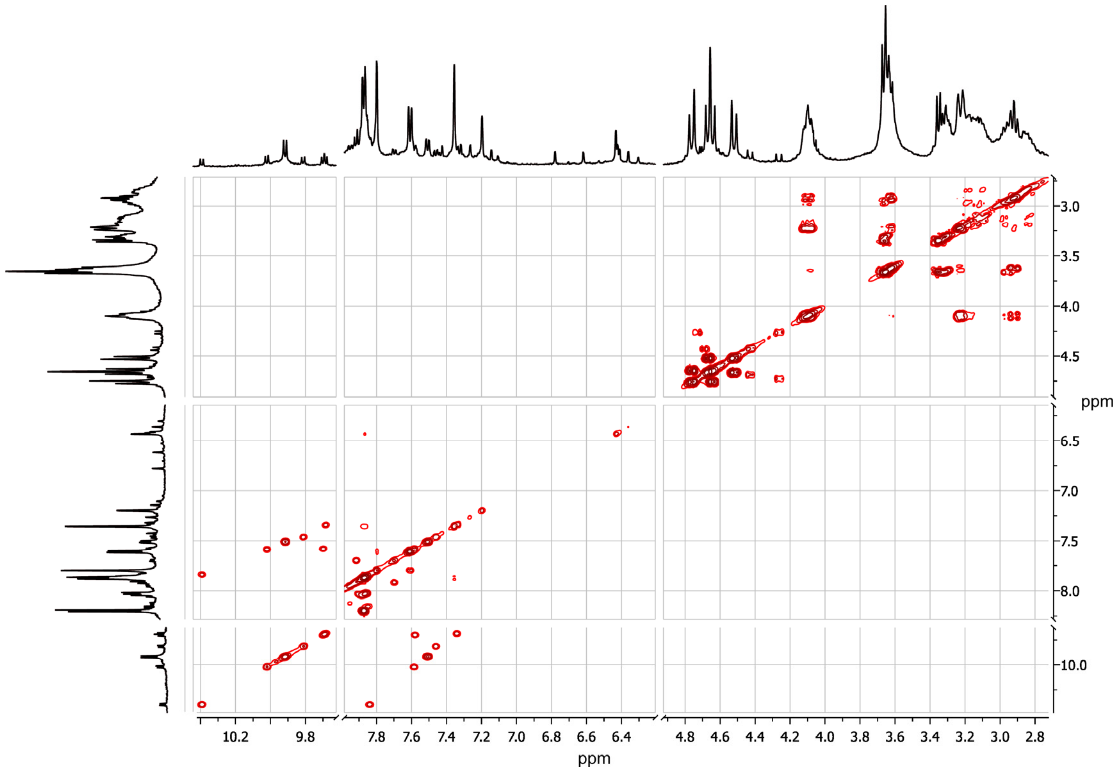

Figure S12. COSY NMR spectrum (500 MHz, CD_3CN) of a mixture of **7**, **8**, **9** and **10**, confirming no coupling between the non-equivalent signals for proton H^A.

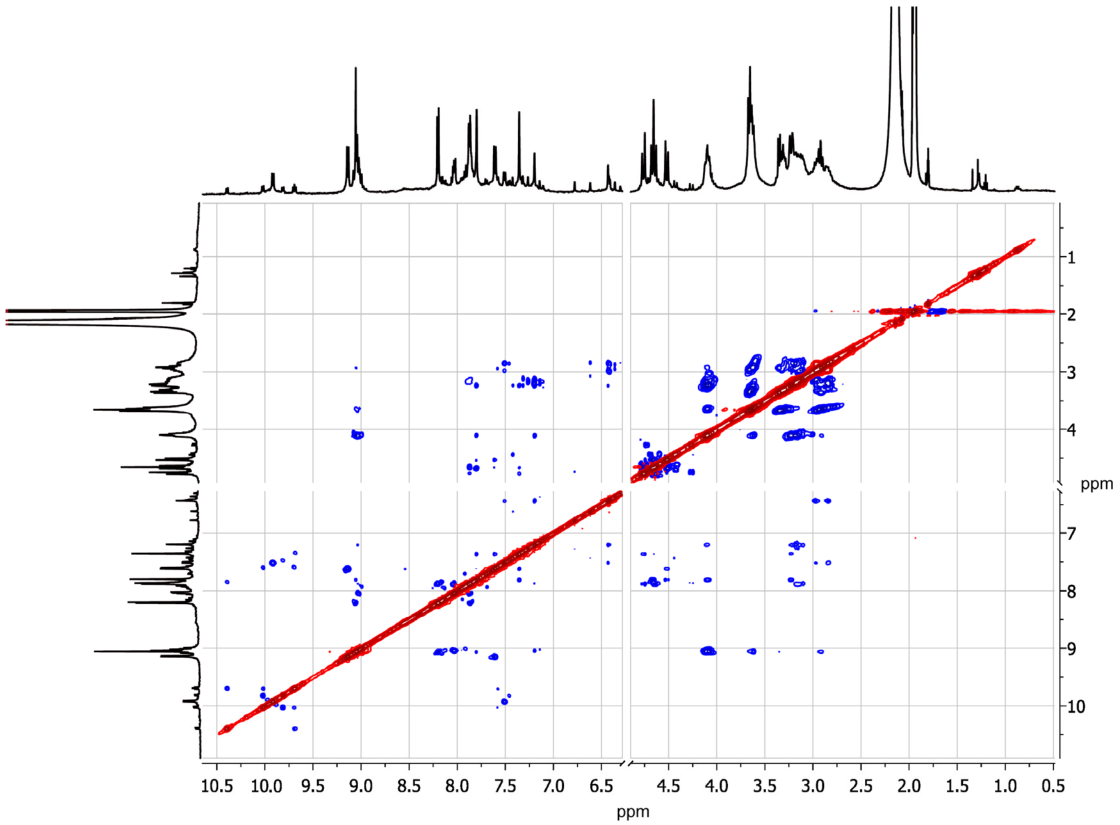

Figure S13. ROESY NMR spectrum (500 MHz, CD_3CN) of a mixture of **7**, **8**, **9** and **10**.

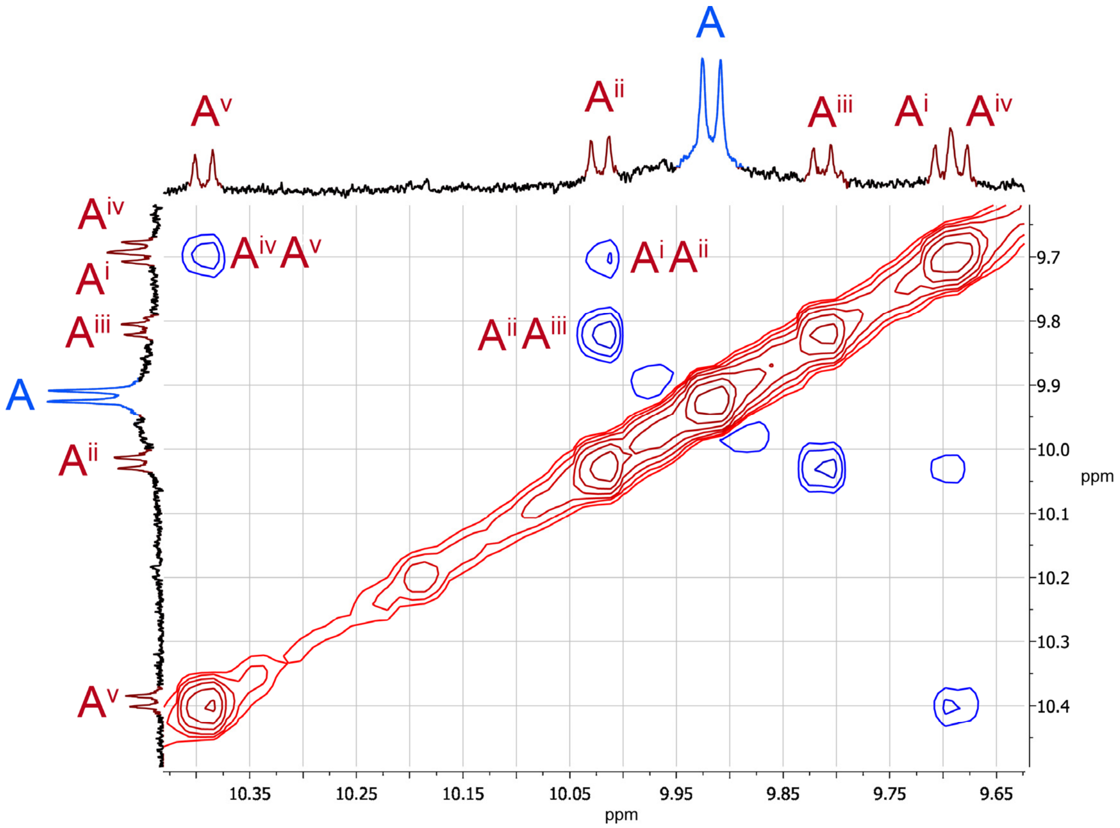

Figure S14. Zoom of ROESY NMR spectrum (500 MHz, CD_3CN) of a mixture of **7**, **8**, **9** and **10** highlighting the region between 9.60 and 10.40 ppm. Cross peaks from mixed-ligand pentamer **9** are shown, consistent with the proposed structure.

2.2.2 Experimental Procedure to Determine the Effects of Mixing on the Relative Yields of Solomon Link 7 and Pentafoil Knot 8

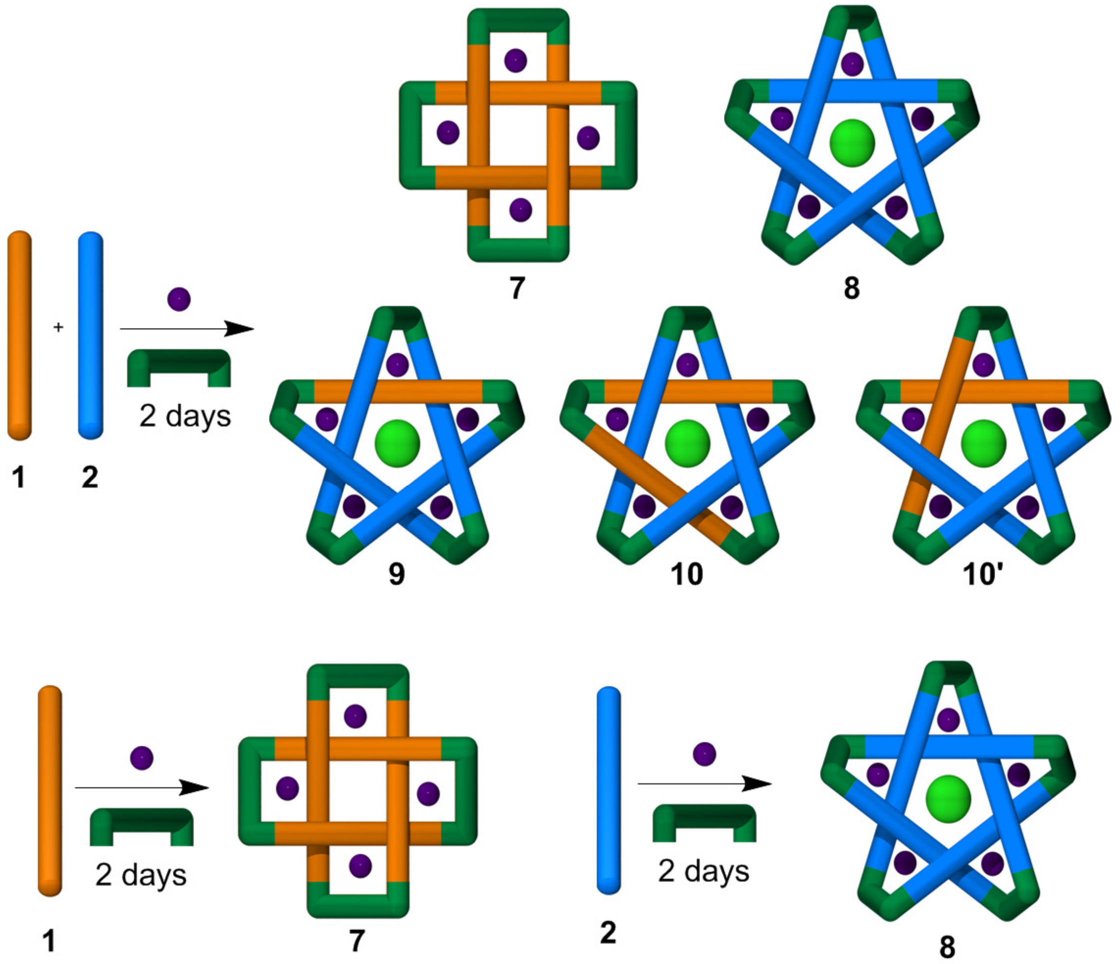

Scheme S5. Self-sorting experiments conducted to determine the effect of mixing on the relative yields of **7** and **8**. Reactions were carried out using stock solutions of dialdehydes **1** and **2** under identical conditions.

To two separate DMSO-d_6 solutions of **1** (2.1 mg, 4.7 μmol, in 1.0 mL) and **2** (2.0 mg, 4.7 μmol in 1.0 mL) was added $FeCl_2$ (50 μL of a 0.10 M stock solution, 5.2 μmol). The two purple solutions were mixed thoroughly to ensure complete dissolution of both dialdehydes. A 0.5 mL aliquot of **1**.$FeCl_2$ was mixed with a 0.5 mL aliquot of **2**.$FeCl_2$ in a clean NMR tube before addition of 2,2'-(ethylenedioxy)bis(ethylamine) **6** (50 μL of a 0.10 M stock solution, 5.2 μmol). The remaining 0.5 mL aliquots of **1**+$FeCl_2$ and **2**+$FeCl_2$ were separately reacted with 2,2'-(ethylenedioxy)bis(ethylamine) **6** (25 μL of a 0.10 M stock solution, 2.6 μmol, for each reaction). The three solutions were heated for 4d at 60 ºC before being allowed to cool. The following purification procedure was applied to each sample individually: The reaction mixture was precipitated using KPF_6 (saturated aqueous

solution). The resulting purple powder was collected on Celite and washed with water, a small amount of EtOH and finally Et_2O. The product mixture was dissolved in CH_3CN and the solvent removed under reduced pressure. The three samples were dissolved in 0.5 mL of CD_3CN and compared by ^1H NMR (Figure S15).

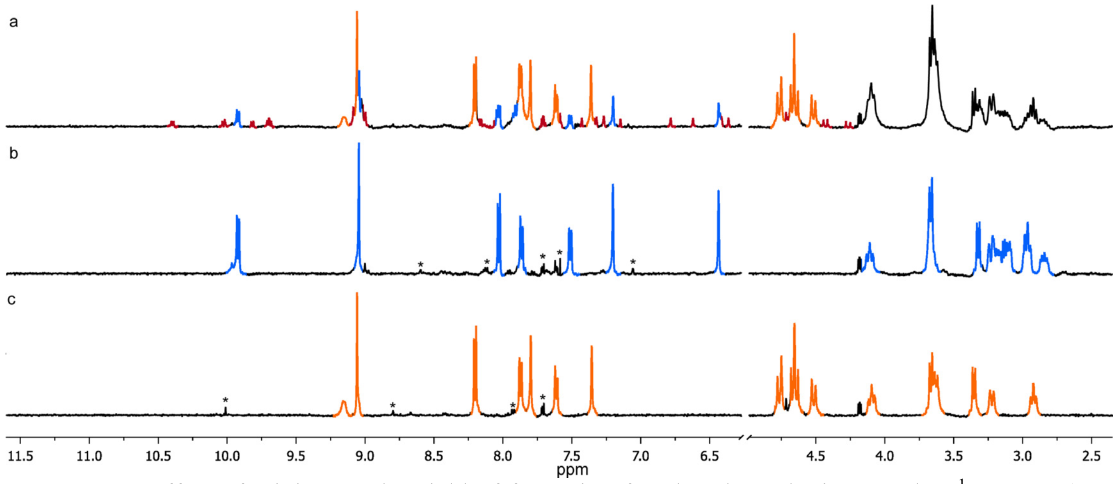

Figure S15. Effect of mixing on the yield of formation for closed topologies **6** and **7**. ^1H NMR (500 MHz, CD_3CN), (a) mixture of **6** (orange), **7** (blue), **8** (red) and **9** (not detected by ^1HNMR, see main text), (b) **7** only, (c) **6** only. * indicates peaks corresponding to aldehyde **1** or **2** resulting from hydrolysis of the respective helicate. Reactions were run at the same concentration with dialdehydes taken from a stock solution. As can be seen the yield of formation of Solomon link **6** is unaffected by the presence of **7** whereas the yield of **7** is drastically reduced with the presence of mix species **8** clearly visible. The total yield of pentafoil knot formation (**7** + **8** + **9**) remains comparable with the yield of **7** in spectrum (b).

2.2.3 Experimental Procedure to Determine the Effects of Concentration on the Self-Sorting of Closed Topologies 7, 8 9 and 10

To two separate DMSO-d_6 solutions of **1** (2.0 mg, 4.7 μmol, in 1.7 mL) and **2** (2.1 mg, 4.7 μmol in 1.7 mL) was added $FeCl_2$ (25 μL of a 0.2 M stock solution, 5.2 μmol). The two purple solutions were mixed thoroughly to ensure complete dissolution of both dialdehydes. Three mixed samples were analyzed differing in the concentration of the reaction mixture.

Sample 4, concentration 5.9 mM:

A 0.5 mL aliquot of **1**+$FeCl_2$ (1.2 μmol of **1**) was mixed with a 0.5 mL aliquot of **2**+$FeCl_2$ (1.2 μmol of **2**) in a clean NMR tube before addition of 2,2'-(ethylenedioxy)bis(ethylamine), **6**, (20 μL of a 0.12 M stock solution, 2.4 μmol).

0.5 mL aliquots of **1**+$FeCl_2$ and **2**+$FeCl_2$ were separately reacted with 2,2'-(ethylenedioxy)bis(ethylamine) **6** (10 μL of a 0.12 M stock solution, 1.2 μmol, for each reaction).

Sample 5, concentration 4.7 mM:

A 0.5 mL aliquot of **1**+$FeCl_2$ was mixed with a 0.5 mL aliquot of **2**+$FeCl_2$ in a clean NMR tube before addition of DMSO-$d_{6,}$ (0.5 mL) then 2,2'-(ethylenedioxy)bis(ethylamine) **6** (20 μL of a 0.12 M stock solution, 2.4 μmol).

0.5 mL aliquots of **1**+$FeCl_2$ and **2**+$FeCl_2$ were separately diluted with DMSO-d_6 (0.25 mL) then reacted with 2,2'-(ethylenedioxy)bis(ethylamine) **6** (10 μL of a 0.12 M stock solution, 1.2 μmol, for each reaction).

Sample 6, concentration 2.4 mM:

A 0.5 mL aliquot of **1**+$FeCl_2$ was mixed with a 0.5 mL aliquot of **2**+$FeCl_2$ in a clean NMR tube before addition of DMSO-$d_{6,}$(1.0 mL) then 2,2'-(ethylenedioxy)bis(ethylamine) **6** (20 μL of a 0.12 M stock solution, 2.4 μmol.

0.5 mL aliquots of **1**+$FeCl_2$ and **2**+$FeCl_2$ were separately diluted with DMSO-d_6 (0.5 mL) then reacted with 2,2'-(ethylenedioxy)bis(ethylamine) **6** (10 μL of a 0.12 M stock solution, 1.2 μmol, for each reaction).

All nine reactions were heated for 2d at 60 ºC before being allowed to cool. The following purification procedure was applied to each sample individually: The reaction mixture was precipitated using KPF_6 (saturated aqueous solution). The resulting purple powder was collected on Celite and washed with water, a small amount of EtOH and finally Et_2O. The product mixture was dissolved in CH_3CN and the solvent removed under reduced pressure. The nine samples were dissolved in 0.5 mL of CD_3CN and compared by ^{1}H NMR (Sample 4, Figure S16. Sample 5, Figure S17. Sample 6, Figure S18).

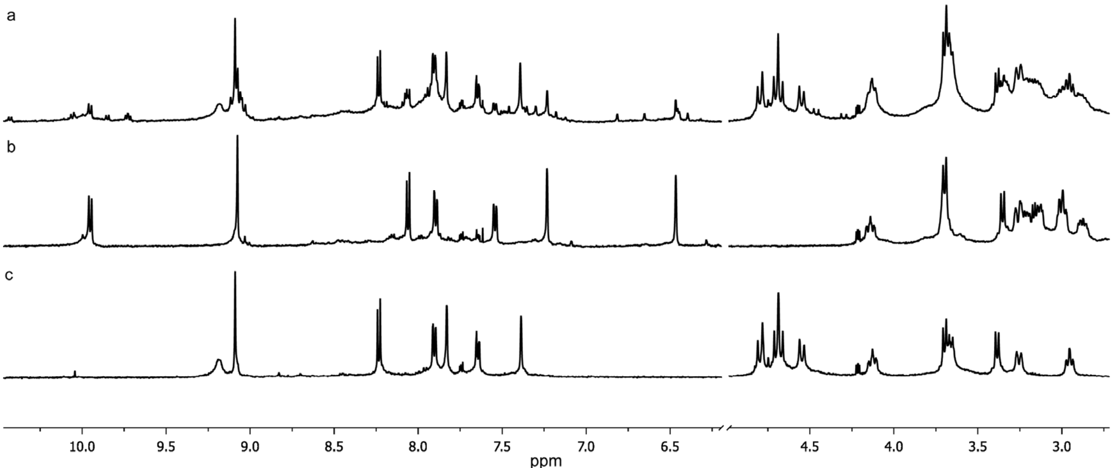

Figure S16. ^{1}H NMR (CD_3CN, 500 MHz) of sample 4 reaction concentration; 5.9 mM. (a) a mixture of **7**, **8**, **9** and **10** (b) **8** and (c) **7**.

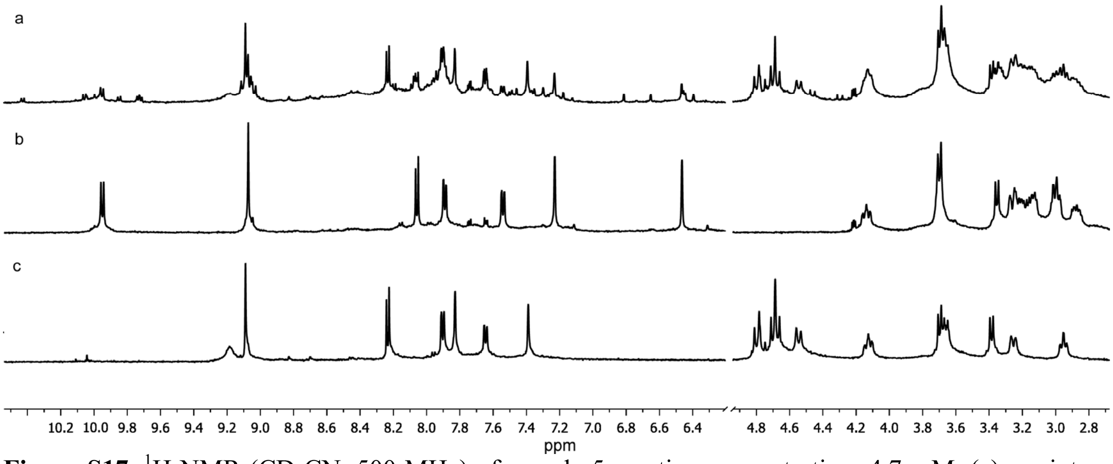

Figure S17. ^{1}H NMR (CD_3CN, 500 MHz) of sample 5 reaction concentration; 4.7 mM. (a) a mixture of **7**, **8**, **9** and **10** (b) **8** and (c) **7**.

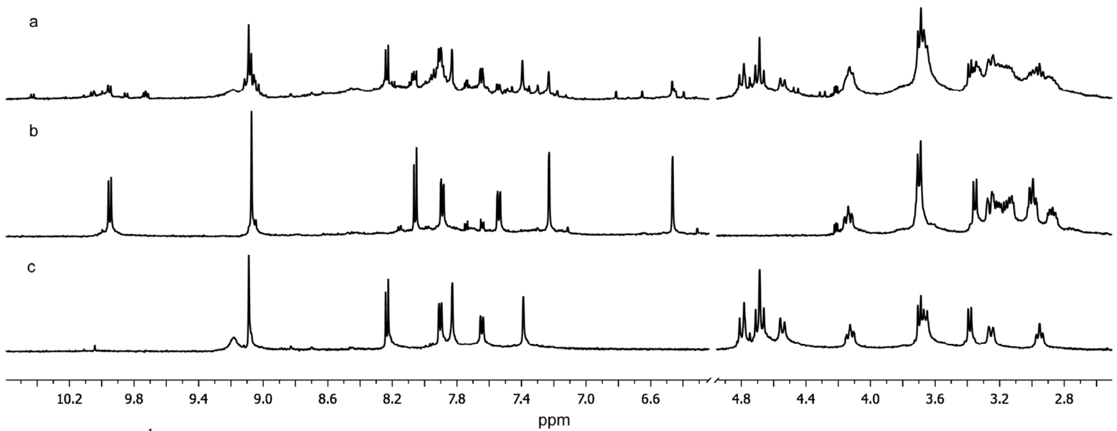

Figure S18. ^{1}H NMR (CD_3CN, 500 MHz) of sample 6 reaction concentration; 2.4 mM. (a) a mixture of **7**, **8**, **9** and **10** (b) **8** and (c) **7**,

2.2.4 Experimental Procedure For the Thermodynamic Investigations of the Self-Sorting of Closed Topologies 7, 8, 9 and 10

See the main text for further details.

To two separate DMSO-d_6 solutions of **1** (2.1 mg, 4.7 μmol, in 1.0 mL) and **2** (2.0 mg, 4.7 μmol in 1.0 mL) was added $FeCl_2$ (50 μL of a 0.10 M stock solution, 5.2 μmol). The two purple solutions were mixed thoroughly to ensure complete dissolution of both dialdehydes. A 0.5 mL aliquot of **1**+$FeCl_2$ was mixed with a 0.5 mL aliquot of **2**+$FeCl_2$ in a clean NMR tube before addition of 2,2'-(ethylenedioxy)bis(ethylamine) **6** (50 μL of a 0.10 M stock solution, 5.2 μmol). The remaining 0.5 mL aliquots of **1**+$FeCl_2$ and **2**+$FeCl_2$ were separately reacted with 2,2'-(ethylenedioxy)bis(ethylamine) **6** (25 μL of a 0.10 M stock solution, 2.6 μmol, for each reaction). The three solutions were heated for 1 d at 60 ºC. The solutions of **1**+$FeCl_2$+**6** and **2**+$FeCl_2$+**6** were combined in a clean NMR tube and thoroughly mixed before being heated at 60 °C for a further 7 days. Both samples were monitored over time (Figures S19 and S20). The two samples were allowed to cool and the following purification procedure was applied to each sample individually: The reaction mixture was precipitated using KPF_6 (saturated aqueous solution). The resulting purple powder was collected on Celite and washed with water, a small amount of EtOH and finally Et_2O. The product mixture was dissolved in CH_3CN and the solvent removed under reduced pressure. Both samples were dissolved in 0.5 mL of CD_3CN and compared by ^{1}H NMR (Figure 3, main text).

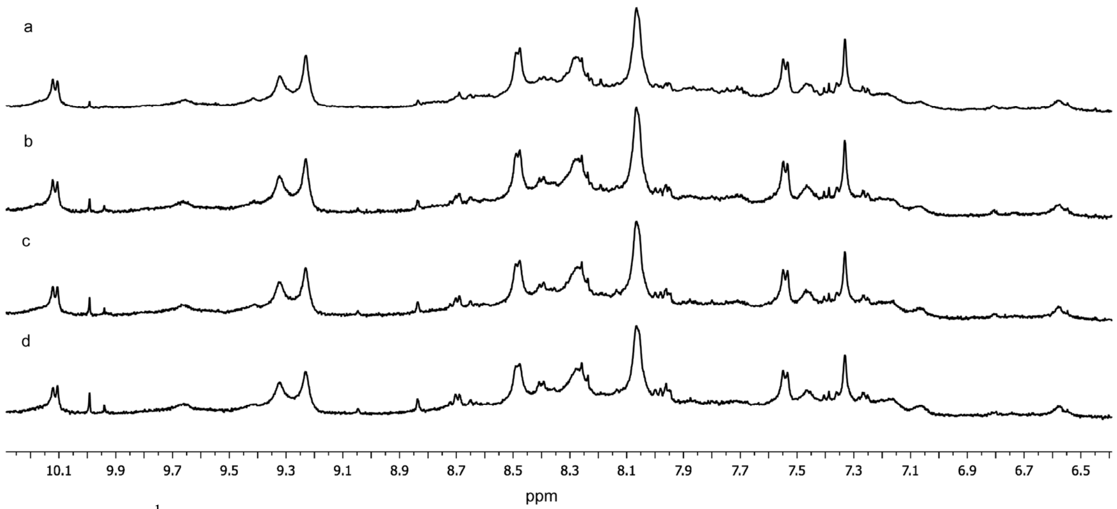

Figure S19. ^{1}H NMR (DMSO-d_6, 500 MHz) of sample A over time, spectra were recorded after heating for (a) 1 days, (b) 2 days, (c) 3 days and (d) 7 days.

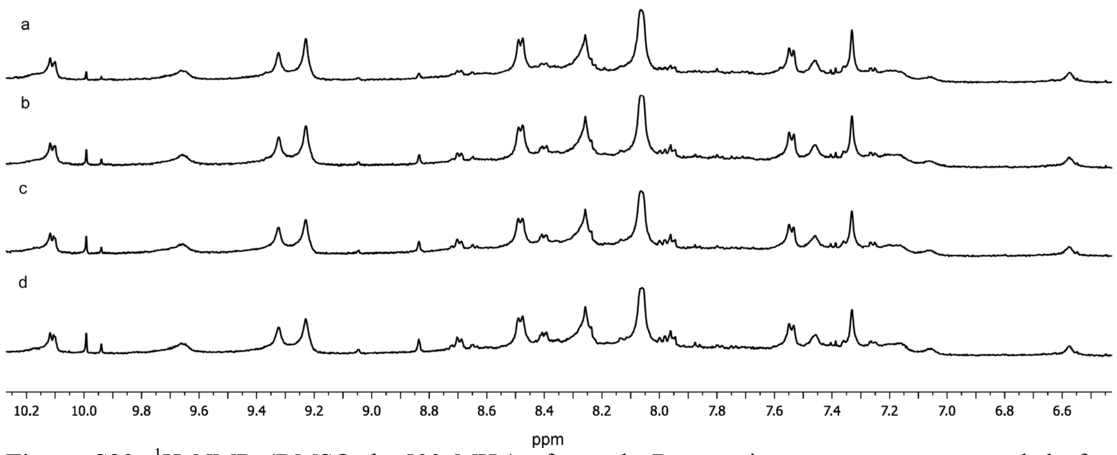

Figure S20. ^{1}H NMR (DMSO-d_6, 500 MHz) of sample B over time, spectra were recorded after mixing then heating for (a) 1 days, (b) 2 days, (c) 3 days and (d) 7 days.

3. References

[S1] J. E. Beves, C. J. Campbell, D. A. Leigh, R. G. Pritchard, *Angew. Chem. Int. Ed.* **2013**, 52, 6464.

[S2] a) J.-F. Ayme, J. E. Beves, D. A. Leigh, R. T. McBurney, K. Rissanen, D. Schultz, *Nat. Chem.* **2012**, *4*, 15; b) J.-F. Ayme, J. E. Beves, D. A. Leigh, R. T. McBurney, K. Rissanen, D. Schultz, *J. Am. Chem. Soc.* **2012**, *134*, 9488.
